# Editorial: Impact of Cancer Plasticity on Drug Resistance and Treatment in Solid Tumors

**DOI:** 10.3389/fonc.2020.596963

**Published:** 2020-10-23

**Authors:** Pascale A. Cohen, Chang Zou, Dong-Hua Yang

**Affiliations:** ^1^Université Lyon 1, Lyon, France; ^2^INSERM, UMR1033 LYOS, Lyon, France; ^3^CRCL-Centre de Recherche en Cancérologie de Lyon-Inserm U1052-CNRS U5286, Lyon, France; ^4^Clinical Medical Research Center, Shenzhen People's Hospital, Jinan University, Shenzhen, China; ^5^Department of Pharmaceutical Sciences, College of Pharmacy and Health Sciences, St. John's University, Queens, NY, United States

**Keywords:** cancer plasticity, solid tumor, drug resistance, biomarker, therapeutic

The Research Topic “Impact of Cancer Plasticity on Drug Resistance and Treatment in Solid Tumors” consists of 32 articles contributed by more than 270 authors in the field of oncology, pharmacology, and translational research. Our aim was to provide a collaborative discussion on molecular and cellular regulators of cancer cell plasticity contributing to tumor progression and drug resistance for the future direction of biomarker discovery and therapeutic strategies.

Cancer stem cells, tumor microenvironment, stroma/cancer cells interactions, changes in metabolism and epithelial-mesenchymal transition offer explanation for tumor plasticity. The current state of art in this era was elegantly reviewed by Fanelli et al., Yang et al., and Lin X. et al., who discussed the clinical relevance of cancer cell plasticity, the novel approaches for monitoring tumor plasticity and the current advances for therapeutic targeting. Yu et al. found that the FAP-a^+^GOLPH3^+^ immunophenotype, combining the expression of both the fibroblast activation protein-alpha and the oncogenic Golgi phosphoprotein 3 protein predict the recurrence and progression of ductal carcinoma *in-situ* (DCIS) into invasive breast cancer. Yao et al. demonstrated in mouse experiments that the levels of MTA3 and SOX2 decreased and increased, respectively, during the progression of tongue squamous cell cancer (TSCC), and that MTA3^low^/SOX2^high^ can serve as an independent prognostic factor for TSCC patients. Chen et al. confirmed that overexpression of PD-L1 occurred predominantly in highly aggressive glioma cells, and Akt binding/activation prevented autophagic cytoskeleton collapse, thus facilitating glioma cell invasion upon starvation stress. Sun et al. showed that SIRT5, a mitochondrial class III NAD-dependent deacetylase, contributes to cisplatin resistance in ovarian cancer by suppressing cisplatin-induced DNA damage in a reactive oxygen species (ROS)-dependent manner, via the regulation of the nuclear factor erythroid 2-related factor 2 (Nrf2)/heme oxygenase 1 (HO-1) pathway. The study from Tang et al. suggested that the Pigment epithelium-derived factor (PEDF) participates the carcinogenesis of human esophageal squamous cell carcinoma and might be a candidate therapeutic target. Finally, analyses conducted by Zhang J. et al. on single-cell sequencing datasets of several human cancers indicated a tumor suppression function of the ZNF671 transcription factor. Fahs et al. demonstrated that the PAX3-FOXO1 fusion protein modulates exosome cargo to confer a protective effect on recipient cells against oxidative stress and to promote plasticity and survival, potentially contributing to the known aggressive phenotype of the fusion gene-positive subtype of Rhabdomyosarcoma. Guo T. et al. reported a clinical case showing change of pathological type to metaplastic squamous cell carcinoma of the breast during disease recurrence.

Epigenetic reprogramming favors cancer plasticity. The discovery of non-coding RNA such as microRNA (miR), Long non-coding RNA (LncRNA) and circular RNA (circ-RNA) is propelling the future advancement of biomarker development and offers opportunities to understand their role in the hallmarks of cancer, including signaling pathways involved in cell proliferation, cell invasion, metabolic plasticity and drug resistance. Wan et al. deciphered the functional domains of the channel-kinase transient receptor potential ion channel subfamily M, member 7 (TRPM7) involved in glioma cell growth or migration/invasion. TRPM7 was found to regulate miR-28-5p expression, which suppresses cell proliferation and invasion in glioma cells by targeting the Rap1b signaling. Guo J. et al. demonstrated that miR-204-3p, whose down-regulation was significantly associated with poor prognosis in bladder cancer patients, negatively modulated the proliferation of bladder cancer cells via targeting the lactate dehydrogenase (LDHA)-mediated glycolysis. Huang et al. elegantly provided evidence that LncRNA AFAP1-S1 up-regulates the RRM2 protein levels by sponging miR-139-5, then activating an RRM2/EGFR/Akt axis that promotes chemoresistance in non-small cell lung cancer. Supportive *in vivo* experiments further demonstrated that knockdown of AFAP1-AS1 significantly suppressed tumor growth and chemoresistance. Li W. et al. proved that miR-199a, by directly regulating K-RAS and thus the downstream AKT and ERK signaling, inhibits glioma cell proliferation *in vitro*, tumor growth *in vivo* and increases sensitivity to telozomide, a drug used in first line treatment of glioma. Lin X.-J. et al. highlighted the role of miR-936 in sensitizing laryngeal squamous cancer cells to doxorubicin and cisplatin. Liu C. et al. experiments suggested that miR-34a-5p, by directly targeting thymidine kinase 1 (TKI), may be part of the mechanisms negatively regulating TKI-driven thyroid carcinoma cell aggressiveness. Growing body of evidence indicate that circRNAs play a role in disease progression, partly by sponging miRNA, and may be used as biomarkers. Gao et al. identified a candidate circRNA associated with poor prognosis in multiple myeloma. Finally, the review by Guo Q. et al. elegantly depicted and discussed the role of exosomal miRNA as a regulators and biomarkers in cancer drug resistance.

It is of utmost importance to decipher how chronic exposure to environmental carcinogens contribute to cell plasticity and tumor progression. The identification of such molecular mechanisms may help in the discovery of human biomarkers of environmental carcinogen exposure and the development of candidate preventive strategies. Using an *in vitro* model for malignant transformation of normal lung cells upon long-term exposure to cigarette smoke, Wang et al. deciphered complex miRNA-mRNA networks associated with cancer-related signaling pathways, in particular those governing the metastasis-associated epithelial-mesenchymal transition and the PI3K/Akt/mTOR survival pathway. Donini et al. demonstrated a functional interplay between the aryl hydrocarbon receptor (AhR) and the G protein-coupled receptor 30 (GPR30) by which chronic and low-dose exposure of the genotoxic Benzo[a]pyrene and/or the endocrine disruptor Bisphenol A fosters the progression of early-transformed human mammary cells into a more aggressive stage. Zhang F. et al. proved that the chronic exposure of endometrial carcinoma cells to the polybrominated diphenyl ether endocrine disruptor BDE-47 triggers phenotypic plasticity, promotes progression and chemoresistance to cisplatin or paclitaxel, at least in part, via ERα/GPR30 and EGFR/ERK signaling pathways.

Antineoplastic drugs can induce cancer cells resistant to treatment that makes the therapeutic effect reduced. Circumventing drug resistance or delineating novel biomarkers of drug efficacy thus represent a great challenge, in particular in the era of precision medicine. To overcome multidrug resistance (MDR) mediated by overexpression of ATP binding cassette (ABC), Wang et al. provided a promising strategy, by nicely highlighting that NVP-TAE684, a novel ALK inhibitor, could reduce the chemo-resistance of MDR cells via the reversion of ABCG2-mediated efflux activity. Zhou et al. found that Erastin inhibits the drug efflux activity of ABCB1 and reverses ABCB1-mediated docetaxel resistance in ovarian cancer. Altogether, these two studies reveal that combination of NVP-TAE684 or Erastin with classical chemotherapy may offer potential effective combinations for the treatment of MDR cancers. Fan et al. explored novel methods to circumvent MDR in B cell lymphoma and demonstrated that an engineered anti-CD19(Fab)-lidamycin cytotoxic fusion protein could effectively inhibit, both *in vitro* and *in vivo*, the growth of adriamycin-resistant B cell lymphoma cells. Finally, to clarify the correlation between drug efficacy and mutations in circulating tumor DNA (ctDNA), Cao et al. monitored the mutational changes and therapeutic response of late-stage colorectal cancers following chemotherapy combined with bevacizumab and/or cetuximab, and confirmed that dynamic changes in drug resistance can be sensitively monitored by gene variation status in ctDNA.

Elucidating the mechanisms of cancer plasticity offers new opportunities for the development of therapeutic targeting. Among future candidate targets is the microbiota, demonstrated to be involved in both tumor initiation and progression. Zhang H. et al. found that Canmei formula, a classical traditional Chinese herbal formulation, reduced *in vivo* colitis-associated colorectal carcinogenesis by modulating inflammation and the composition of the gut microbiota. Huang et al. explored *in vitro* the anticancerous activity the Methyl-cantharidimide drug, originally discovered in insects, and proved that it inhibits growth of human hepatocellular carcinoma cells by inducing cell cycle arrest and promoting apoptosis. Nan et al. proposed that ROS-mediated proteasome-dependent pathway can be exploited to overcome apoptosis resistance triggered by aberrant expression of the anti-apoptotic survivin protein in cancers, by demonstrating that the alkaloid/amide Piperlongumine extracted from peppers efficiently induced the proteasome-dependent degradation of the survivin *in vitro*, downregulated survivin *in vivo* and inhibition of ovarian cancer cells xenograft tumor growth. Finally, an elegant study from Liu K. et al. identified, by proteomics investigation of breast cancer specimen, increased protein levels of Hsp90 proteins associated with poor prognosis. BJ-B11, an Hsp90 inhibitor, was shown to hamper *in vitro* the cancerous properties of triple negative breast cancer cells and to inhibit tumor growth in xenograft model. Wen et al. explored the antitumor effects of 7-methoxy-1-tetralone in hepatocellular carcinoma and found that this compound induces apoptosis, suppresses cell proliferation and migration via regulating c-Met, p-AKT, NF-κB, MMP2, and MMP9 expression. Finally, Yan and Wang nicely reviewed brain cancer-associated alterations of proteoglycans making these latter as putative biomarkers or therapeutic targets.

In conclusion, the “Impact of Cancer Plasticity on Drug Resistance and Treatment in Solid Tumors” Research Topic highlights the complex phenomenon of cancer cell plasticity. The recent insights into the role of plasticity in cancer progression implies the need to continue improving our understanding into the fundamental mechanisms governing tumor progression and drug resistance, with the aim to identify relevant new biomarkers and to develop innovative therapies.

## Author Contributions

All authors listed have made a substantial, direct and intellectual contribution to the work, and approved it for publication.

## Conflict of Interest

The authors declare that the research was conducted in the absence of any commercial or financial relationships that could be construed as a potential conflict of interest.

